# Improvements in a patient with upper limb double crush syndrome through integrative Korean medicine treatment: A case report

**DOI:** 10.1097/MD.0000000000043745

**Published:** 2025-08-15

**Authors:** So Rim Kim, Sook-Hyun Lee, Yeon Sun Lee, Jin Hyun Kim, Min Jo Seo, Yoon Jae Lee, In-Hyuk Ha

**Affiliations:** a Department of Acupuncture and Moxibustion, Bucheon Jaseng Korean Medicine Hospital, Bucheon, Republic of Korea; b Jaseng Spine and Joint Research Institute, Jaseng Medical Foundation, Seoul, Republic of Korea; c Department of Oriental Neuropsychiatry, Bucheon Jaseng Korean Medicine Hospital, Bucheon, Republic of Korea; d Department of Internal Korean Medicine, Bucheon Jaseng Korean Medicine Hospital, Bucheon, Republic of Korea; e Department of Korean Medicine Rehabilitation, Bucheon Jaseng Korean Medicine Hospital, Bucheon, Republic of Korea.

**Keywords:** case report, cervical radiculopathy, double crush syndrome, Korean medicine treatment, radial nerve entrapment

## Abstract

**Rationale:**

Double crush syndrome (DCS) refers to multi-nerve entrapment based on the hypothesis that the distal nerve is likely to be more affected by pressure in the presence of a proximal lesion. DCS is difficult to diagnose, and thus far, few cases have been diagnosed from the perspective of DCS and reportedly treated with traditional Korean medicine.

**Patient concerns:**

The patient was a 50-year-old Korean man with DCS, hand edema, cervical radiculopathy, and upper limb paraesthesia.

**Diagnoses:**

Based on the characteristic disc symptoms and symptoms extending beyond the dermatome and myotome of the C7/T1 nerve, the patient was diagnosed with DCS.

**Interventions:**

The patient underwent 2 phases of Korean medicine treatment, including acupuncture, pharmacopuncture, Chuna manual therapy, and kinesiology taping, for 2 months.

**Outcomes:**

Significant improvement was noted in the patient’s assessments (neck pain numeric rating scale scores of 7 and 3; handshake test 50% and 90%, Spurling test (++) and (−), range of neck motion (30/20/15/15; 45/45) and (45/45/45/45; 90/90) at initial treatment and end of treatment, respectively; during phase 1, pitting edema grade scale 2 and 1 at initial treatment and end of phase 1, respectively; during phase 2, the tenderness of the lateral epicondyle of the right elbow (++) and (+−), upper limb tension test for median and radial nerve (+) and (+−) at initial treatment of phase 2 and end of phase 2, respectively). At the 3-month follow-up, physical examination findings were either further improved or maintained, and cervical spine magnetic resonance imaging also demonstrated improvement. Since oral pharmacological treatment was not utilized, no blood parameters were assessed beyond the physical examination.

**Lessons:**

Integrative Korean medicine can be considered as a treatment for DCS. More studies on the diagnosis of DCS using ultrasonography and the effectiveness of Korean medical treatment for DCS are needed in the future.

## 1. Introduction

Cervical disc herniation is a common cause of cervical radiculopathy, with an annual incidence of 18.6/1,00,000 individuals, peaking after 60 years of age.^[1]^ Patients with cervical disc herniation and radiculopathy typically report severe neck and upper limb pain.^[2]^ Upper limb pain often follows a myotomal pattern, whereas sensory symptoms, such as burning or tingling, are distributed in a dermatomal pattern. These radicular symptoms can also be associated with altered reflexes and decreased upper limb muscle strength.^[3]^

Nerve entrapment syndrome is neuropathy caused by structural abnormalities (e.g., nerve compression, displacement, or traction) or intrinsic nerve abnormalities (e.g., neuronal tumors).^[4]^ It is essential to distinguish conditions similar to specific nerve entrapment syndromes from cervical radiculopathy-associated with cervical disc herniation.^[5]^ Upton and McComas found that 70% (81 out of 115) of patients with carpal tunnel syndrome or cubital tunnel syndrome also had concurrent cervical nerve damage, coining the expression “double crush syndrome” (DCS) to describe this phenomenon.^[6]^ Proximal lesions increase the vulnerability of distal nerves to compression, resulting in symptoms and complications that exceed expectations based on isolated distal entrapment.^[7]^ Similarly, suboptimal surgical outcomes in patients with carpal tunnel syndrome may stem from multisite nerve entrapment, which must be considered in treatment planning.^[8]^

Traditional Korean medicine (TKM) offers various treatment options for neuropathy, including acupuncture, pharmacopuncture, herbal medicine, dry needling, electroacupuncture, thread-embedding acupuncture, and Chuna manual therapy.^[9]^ Pharmacopuncture is widely recognized as an effective treatment for nerve entrapment syndromes,^[10]^ to maximize the therapeutic effects of acupuncture by administering herbal medicine extracts to meridians and acupoints.^[11]^ A recent study by Lee et al^[9]^ on domestic clinical research trends in pharmacopuncture for neuropathy identified sweet bee venom, bee venom pharmacopuncture, and Shinbaro pharmacopuncture as the most used types known for their anti-inflammatory properties. These treatments typically involve administering pharmacopuncture to tender points or acupoints along nerve pathways.

The DCS hypothesis proposed by Upton and McComas^[6]^ posits that a single nerve lesion can predispose a patient to a second lesion along the same nerve branch. Clinical reports supporting this hypothesis frequently emphasize associations between cervical radiculopathy: thoracic outlet syndrome and distal nerve entrapment syndromes, particularly carpal tunnel syndrome.^[12]^ Although there are numerous studies on DCS in Western medicine (e.g., investigations into differences in peripheral neuropathy with and without DCS)^[13]^ and the concept of multifocal neuropathy,^[14]^ little research has been conducted to address DCS treatment from a Korean perspective.

Although ongoing research has explored the symptoms of cervical disc herniation and Korean medical treatments (e.g., pharmacopuncture) for nerve entrapment, there is a significant lack of research on DCS in Korea. Against this backdrop, this study introduces DCS through a case report and evaluates its treatment effects from a TKM perspective.

## 2. Case presentation

This is a retrospective report of a patient with DCS who received outpatient treatment at our hospital for 8 weeks (October 27–December 20, 2023). This study was approved by the Institutional Review Board of Jaseng Korean Medicine Hospital (approval number: JASENG 2024-04-007). The treatments described below were performed and evaluated by 2 TKM doctors.

### 2.1. Patient information

A 50-year-old Korean man with no relevant family history was diagnosed with diabetes in 2021. He presented to our hospital with pain radiating from the right posterior cervical region to the trapezius and medial scapular border, along with paraesthesia extending from the right forearm to the fingers. These symptoms developed after he was hit by a heavy object near the thoracic spine and scapular area while moving loads on October 13, 2023. The patient underwent 10 days of outpatient treatment at a local pain clinic and 2 days of treatment at a local TKM clinic. However, he continued to experience discomfort in the right scapula and thoracic region and severe tingling and paraesthesia on the underside of his right little finger.

During the first visit, the cervical range of motion (ROM) measurements were as follows: flexion at 30°, extension at 20°, right lateral flexion at 15°, left lateral flexion at 15°, right rotation at 45°, and left rotation at 45°. The pain intensity was rated 7 on a numeric rating scale (NRS). The Spurling test was positive on the right hand, and the right hand showed grade 2 pitting edema on the pitting edema grade scale. A handshake test indicated a right hand grip strength of 50% compared to that with the left hand.

**Figure 1.**
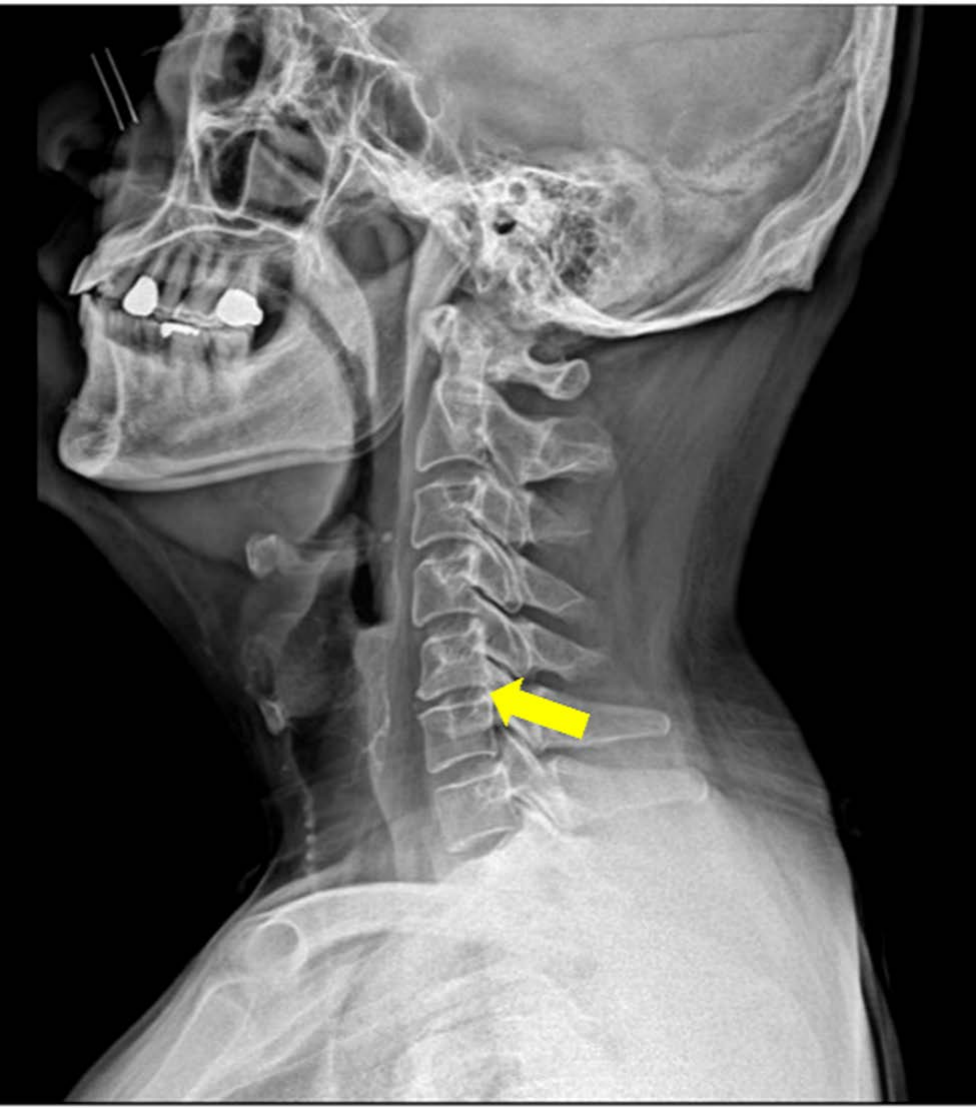
C-spine X-ray (October 27, 2023).

**Figure 2.**
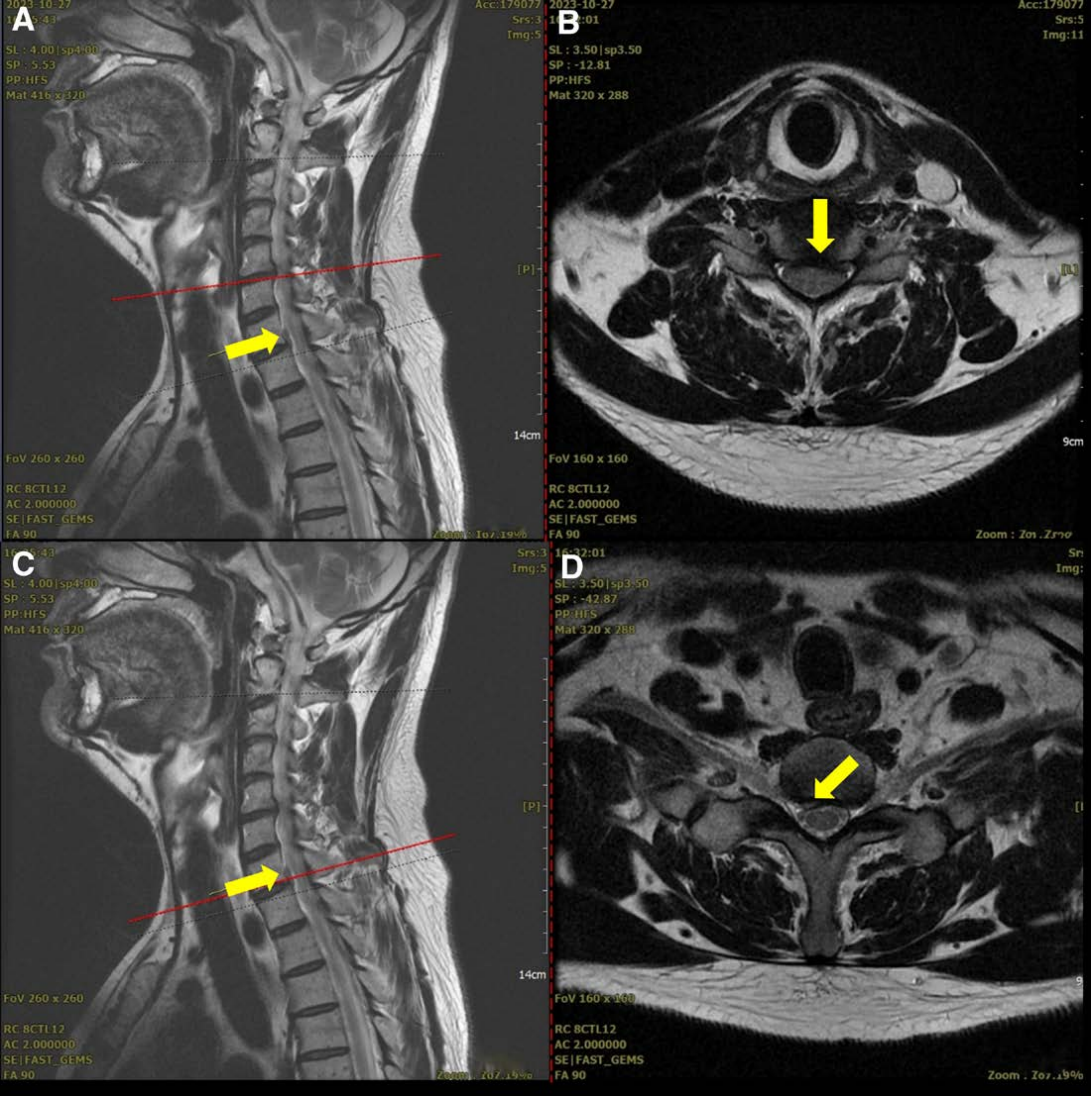
C5/6 magnetic resonance imaging on October 27, 2023 (A, B) and C7/T1 magnetic resonance imaging on October 27, 2023 (C, D).

Cervical spine (C-spine) radiography revealed decreased cervical lordosis and narrowing of the C5/6 disc space (Fig. 1). C-spine magnetic resonance imaging (MRI) showed bilateral neural foraminal stenosis caused by a diffuse, symmetric, and moderately bulging disc at C5/6 (Fig. 2A), along with right central-subarticular focal extrusion and cephalic migration of the disc, compressing the thecal sac and right C8 spinal nerve root at C7/T1 (Fig. 2B).

### 2.2. Treatments

**Figure 3.**
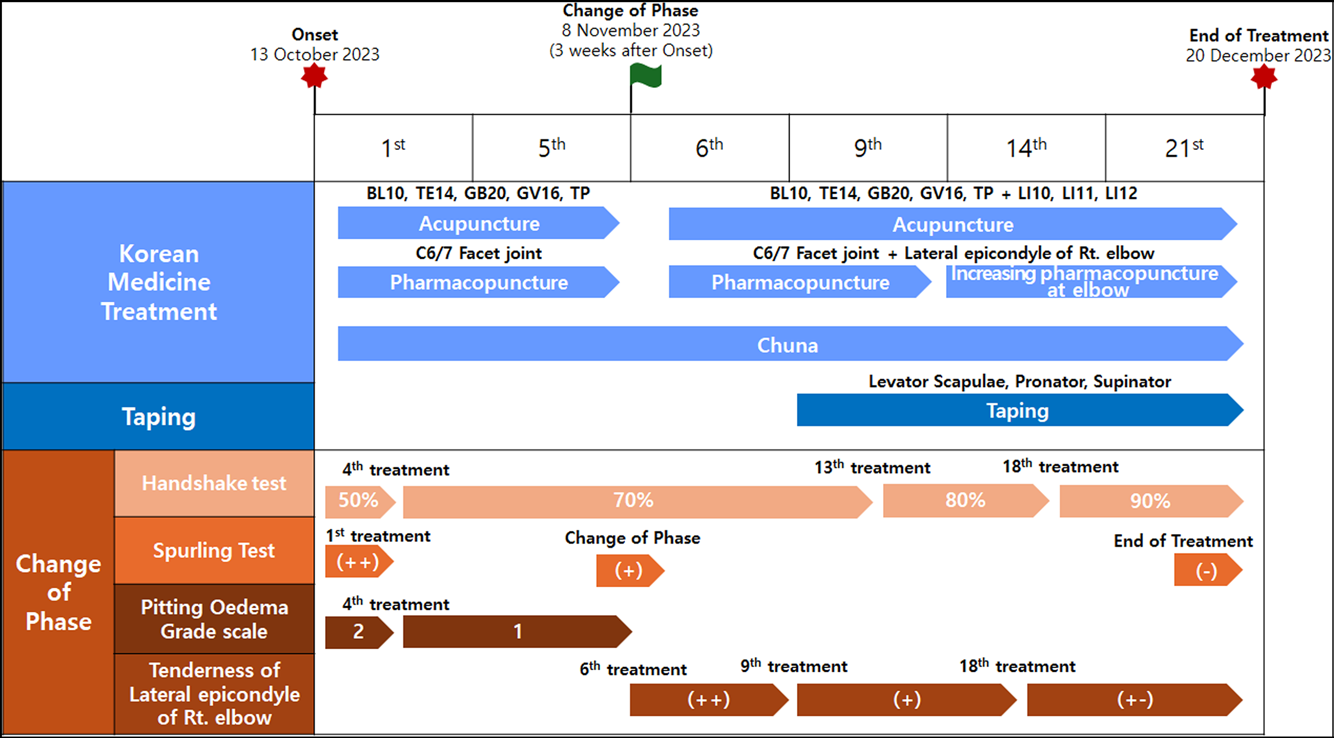
Timeline of integrative traditional Korean medicine treatment and phase change.

The patient exhibited characteristic disc symptoms, including paraesthesia in the fifth digit of the right hand and muscle weakness accompanied by grade 2 pitting edema, a positive upper limb tension test (ULTT), and symptoms extending beyond the dermatome and myotome of the C7/T1 nerve, such as tenderness and pain. Based on these findings, the patient’s condition could not be explained solely by cervical radiculopathy. Therefore, treatment was approached from the perspective of DCS. Patient care was divided into 2 phases: in phase 1 (weeks 1–2), treatment focused on addressing the proximal lesion (i.e., cervical radiculopathy) and focal extrusion of the right central-subarticular region at C7/T1; and in phase 2 (weeks 3–8), treatment continued with a combined focus on the distal lesion (i.e., radial tunnel syndrome). The criteria for transitioning between phases included mild recovery of grip strength, reduction in swelling, changes in Spurling test results, and emergence of trauma and tenderness corresponding to week 3 after onset (Fig. 3).

Sterile disposable stainless steel needles (0.25 mm × 30 mm; Dongbang Acupuncture) were used for acupuncture. Treatments were performed daily, with approximately 15 needles inserted at a depth of 10 to 30 mm and retained for up to 15 minutes. During these sessions, electrical stimulation at 2 Hz and transcutaneous infrared irradiation were administered. In phase 1, needling was performed on the bladder meridian BL10 (Tianzhu), triple energizer meridian TE14 (Jianliao), gall bladder meridian GB20 (Fengchi), governor vessel meridian GV16 (Fengfu), and trigger point (Ashixue) to regulate qi and blood in the cervical and shoulder girdle regions. In phase 2, additional points included the large intestine meridians LI10 (Shousanli), LI11 (Quchi), and LI12 (Zhouliao), as well as tender points near the right lateral epicondyle (Table 1).

**Table 1:** Treatments during the first and second phases.

	First phase	Second phase
Acupuncture	Cervical spine, shoulder girdle BL10, TE14, GB20, GV16, TP	Cervical spine, shoulder girdle + elbow BL10, TE14, GB20, GV16, TP + LI10, LI11, LI2
Pharmacopuncture	C6/7 facet joint 2 mL	C6/7 facet joint 2 mL + right elbow lateral epicondyle 0.5–1 mL
Taping	X	Right elbow, shoulder girdle

BL = bladder meridian (used in acupuncture), BL10, TE14, GB20, GV16, LI10, LI11, LI12 = specific acupuncture points (on various meridians), C6/7 = cervical vertebrae 6 and 7, GB = gall bladder meridian (used in acupuncture), GV = governor vessel meridian (used in acupuncture), LI = large intestine meridian (used in acupuncture), TE = triple energizer meridian (used in acupuncture), TP = trigger point.

Pharmacopuncture was administered once daily with acupuncture using single-use syringes (Sungshim-Insulin Syringe, 1 mL, 29 G × 13 mm; Feeltech-Disposable Syringe, 3 mL, 23 G × 25 mm). Shinbaro pharmacopuncture solution was prepared using the Jaseng External Herbal dispensary. In phase 1, a dose of 2 mL was injected into the C6/7 facet joint. In phase 2, in addition to the phase 1 injection, 0.5 to 1 mL was injected at a tender point near the right lateral epicondyle for a total of 2.5 to 3 mL per session (Table 1).

Taping therapy was introduced during the nineth treatment session, based on the patient’s symptoms and physical examination findings. Temtex kinesiology tape was applied to the levator scapulae, pronator, and supinator muscles as determined by the practitioner’s evaluation.

Chuna manual therapy was performed consistently throughout both phases using identical techniques focused on the C-spine and shoulder girdle. These techniques include the supine position cervical correction method, prone position cervical distraction method, supine position cervical JS (Joint stretching (related to Chuna manual therapy) distraction and correction method, and supine position sternoclavicular correction. Each session lasted approximately 10 minutes and was conducted once daily before or after acupuncture. After each session, a reevaluation was performed to assess displacement correction and pain reduction.

### 2.3. Outcome measures

The patient’s symptoms were evaluated using the NRS, ROM of the C-spine, handshake test, Spurling test, ULTT, pitting edema grade scale, and tenderness of the lateral epicondyle of the right elbow. Given that the treatment involved acupuncture, pharmacopuncture, Chuna manual therapy, and kinesiology taping, and did not include the use of oral medications, blood parameters were not evaluated.

The NRS was used to evaluate overall pain intensity. The patient selected a number representing his perceived pain level, usually on an 11-point scale (NRS-11) ranging from 0 to 10.^[15]^ The pain was assessed before each outpatient treatment session.

ROM assesses the active ROM within a pain-free range. For the C-spine, the active ROM was measured in 4 directions: flexion, extension, lateral bending (right/left), and rotation (right/left). Measurements were taken at 3 key points: the initial outpatient visit, phase transition, and completion of treatment.

The handshake test is a simple physical examination to assess grip strength without requiring a device. The grip strength was estimated based on the force applied during the handshake. This simple test allows practitioners to objectively assess function, pain, and disease behavior without a formal survey.^[16]^ The handshake test was performed before each outpatient treatment.

The Spurling test is a diagnostic method that involves extending the neck and rotating the head to the affected side. Localized pain may indicate compression of the intervertebral foramen, suggesting cervical disc herniation, whereas radiating pain indicates nerve root compression.^[17]^ Measurements were taken at 3 key points: the initial outpatient visit, phase transition, and treatment completion.

In the ULTT, brachial plexus or cervical radiculopathy was suspected if pain or neurological symptoms appeared on the affected side in specific test positions (Table 2). Beyond evaluating radiculopathy, the test is also useful for positioning the upper limb nerves at maximum tension to help reduce tension and alleviate symptoms.^[17]^ Measurements were taken before each outpatient treatment in phase 2.

**Table 2:** Position of the upper limb tension test.

Upper limb tension test		
Nerve	Method	
Median nerve	Fix the patient’s clavicle and scapula	Shoulder joint – 90° abduction, external rotation Elbow joint – extension Wrist joint – extension Forearm – supination
Radial nerve		Shoulder joint – 10° abduction, internal rotation Elbow joint – extension Wrist joint – flexion
Ulnar nerve		Shoulder joint – 90° abduction Wrist joint – extension Forearm – pronation Elbow joint – rotation (full) Shoulder joint – external rotation

The pitting edema grade scale was used to evaluate the severity of pitting edema using a 4-point scale based on the pit depth (in mm) and rebound time. Pressure was applied to the assessment site for 5 seconds, and the resulting measurements were recorded.^[18]^ Evaluations were performed after each outpatient treatment during phase 1.

Tenderness of the lateral epicondyle of the right elbow is a specific finding on physical examination indicative of radial tunnel syndrome, which typically presents as pain located 3 to 6 cm distal to the radial head. To assess clinical significance, the examiner compared the tenderness at the affected site with that on the unaffected side.^[9]^ This was evaluated before each outpatient treatment in phase 2.

### 2.4. Follow-up and outcome

**Figure 4.**
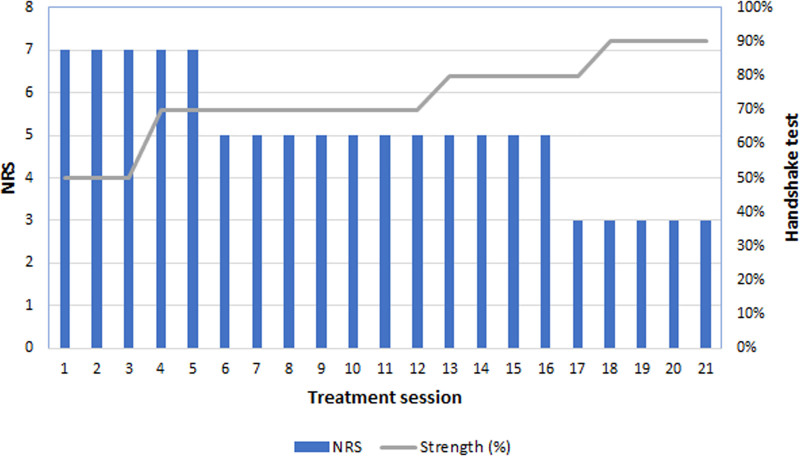
Changes in the daily numeric rating scale (NRS) score and strength were measured using a handshake test. NRS = numeric rating scale.

On October 13, 2023, before the initial outpatient treatment, the patient’s neck pain and paraesthesia in the right forearm and fingers were rated 7 on the NRS. This improved to 5 before the sixth treatment session (the end of phase 1), further decreased to 3 before the 17th session, and was maintained at this level through the end of the treatment. Grip strength, as assessed using the handshake test, was 50% that of the unaffected side before treatment. It improved to 70% before the fourth session, 80% before the 13th session, and 90% before the 18th session, and it was maintained at this level through the end of treatment (Fig. 4).

**Figure 5.**
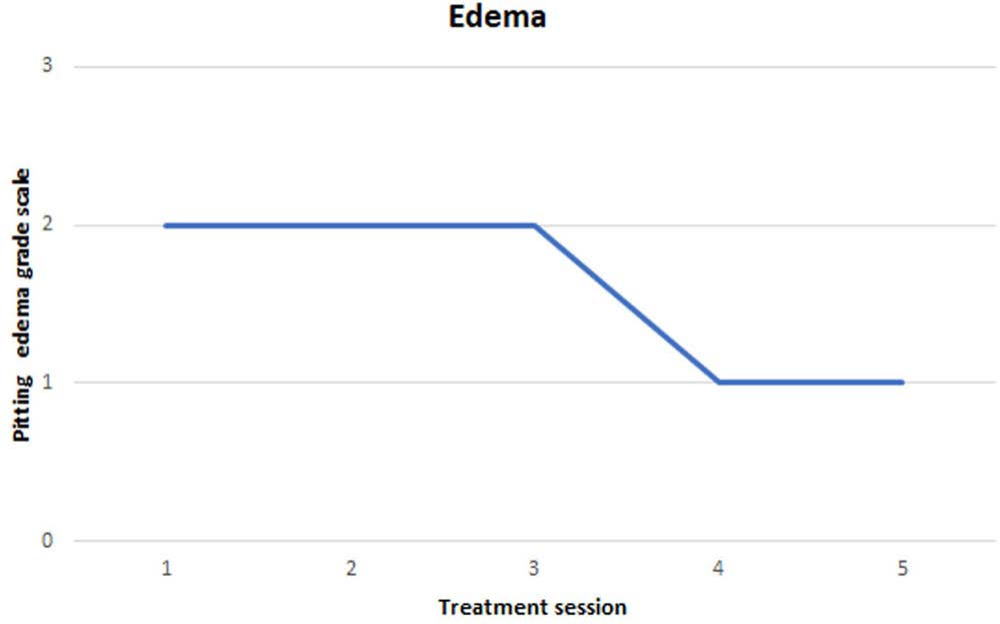
Changes in pitting edema grade scale during phase 1.

The results of the assessments conducted during phases 1 and 2 are reported separately for each phase. In phase 1, the pitting edema improved from grade 2 at the initial outpatient visit to grade 1 after the fifth treatment session (Fig. 5).

In phase 2, the tenderness of the lateral epicondyle of the right elbow improved from (++) before the sixth session (start of phase 2) to (+) before the nineth session (third treatment in phase 2) and then to (+−) before the 18th session (12th treatment in phase 2), and this level was maintained until the end of treatment (Table 3). Regarding the ULTT (assessed only in phase 2), negative findings were consistently observed for the ulnar nerve throughout treatment. For the median nerve, the findings improved from (+) before the sixth session to (+−) before the nineth session, became negative before the 13th session, and remained at this level until the end of the treatment. For the radial nerve, the findings improved from (+) before the sixth session to (+−) before the nineth session and remained at this level until the end of treatment (Table 3).

**Table 3:** Changes in the tenderness of lateral epicondyle and upper limb tension test during the second phase.

Time point	Tenderness of lateral epicondyle of right elbow	ULTT (median nerve)	ULTT (radial nerve)	ULTT (ulnar nerve)
Sixth	(++)	(+)	(+)	(−)
Seventh	(++)	(+)	(+)	(−)
Eighth	(++)	(+)	(+)	(−)
Nineth	(+)	(+−)	(+−)	(−)
10th	(+)	(+−)	(+−)	(−)
11th	(+)	(+−)	(+−)	(−)
12th	(+)	(+−)	(+−)	(−)
13th	(+)	(−)	(+−)	(−)
14th	(+)	(−)	(+−)	(−)
15th	(+)	(−)	(+−)	(−)
16th	(+)	(−)	(+−)	(−)
17th	(+)	(−)	(+−)	(−)
18th	(+−)	(−)	(+−)	(−)
19th	(+−)	(−)	(+−)	(−)
20th	(+−)	(−)	(+−)	(−)
21st	(+−)	(−)	(+−)	(−)

ULTT = upper limb tension test.

The Spurling test findings improved from (++) before the initial treatment to (+) during the phase transition and to (−) at the end of the treatment. The ROM of the C-spine increased from (30/20/15/15; 45/45) at the initial visit to (40/30/30/30; 60/60) during the phase transition and normalized to (45/45/45/45; 90/90) at the end of treatment (Table 4).

**Table 4:** Changes in the Spurling test and range of motion of the C-spine.

Time point	Day 1	Changing of phase	End of treatment
Spurling test	(++)	(+)	(−)
C-spine ROM (º)			
Flexion	30	40	45
Extension	20	30	45
Lateral bending	15/15	30/30	45/45
Rotation	45/45	60/60	90/90

C-spine = cervical spine, ROM = range of motion.

**Figure 6.**
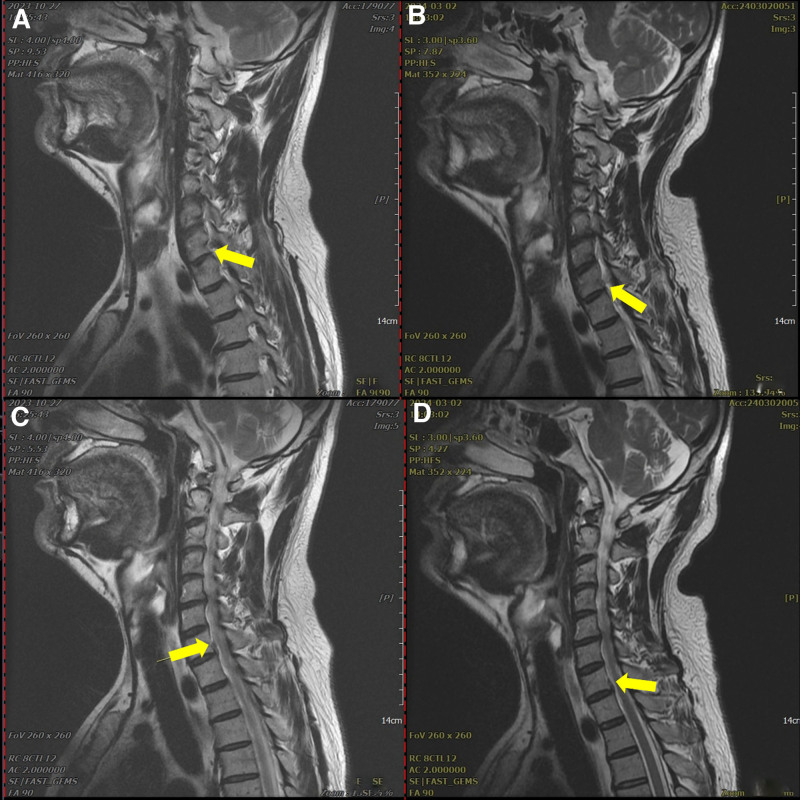
Follow-up of C-spine magnetic resonance imaging on October 27, 2023 (A, B) and March 2, 2024 (C, D).

At a follow-up visit on February 24, 2024, 3 months after the final treatment on December 20, 2023, the Spurling test, ULTT, and tenderness of the lateral epicondyle were negative, and grip strength recovered to 95% to 100%. A follow-up C-spine MRI performed on March 2, 2024 revealed a slight improvement in the acute disc condition at C7/T1 (Fig. 6).

Adherence was confirmed by treatment reservation details. The patient attended 21 out of 26 (80.7%) scheduled acupuncture sessions for 8 weeks. Tolerability was assessed at each outpatient visit through patient’s interviews. Based on the interviews and physical feedback, adjustments were made to the dosage of pharmacopuncture and the duration of taping application as needed. There were no adverse and unanticipated events.

At the follow-up visit, the patient shared his perspective regarding the entire course of treatment:

“When the symptoms first appeared, the edema in my hand and paraesthesia caused significant discomfort. As treatment progressed, the edema subsided, and particularly the taping therapy helped compensate for the weakened muscle strength, which greatly contributed to resuming daily activities. Although I still experience some exacerbation of pain when overexerting myself since the treatment ended in December, the pain is now well managed and incomparable to what I initially experienced. I am highly satisfied with the treatment.”

## 3. Discussion

The patient was a 50-year-old man who presented with pain radiating from the right posterior cervical region to the trapezius and medial scapular border, along with paraesthesia extending from the right forearm to the fingers, following an incident on October 13, 2023, in which a heavy object fell near the thoracic spine and right scapular area while carrying loads. At the initial visit, the patient reported discomfort around the right scapula and thoracic spine, tingling pain, and paraesthesia extending from the right forearm to the underside of the little finger. Examination revealed limited ROM (30/20/15/15; 45/45), Spurling test results of (−/+), grade 2 pitting edema in the right hand, positive ULTT, tenderness over the right lateral epicondyle, and grip strength at 50% compared with the measurements on the unaffected side. Imaging studies revealed reduced cervical lordosis, narrowing at C5/6 on C-spine radiography, and stenosis with a moderately bulging disc at C5/6 and right central-subarticular focal extrusion at C7/T1 on C-spine MRI.

The patient showed concurrent or sequential symptoms of cervical radiculopathy (paraesthesia in the fifth digit of the right hand and muscle weakness corresponding to the nerve distribution) and peripheral nerve entrapment (hand edema, positive ULTT findings, and tenderness). During the last half of phase 1, improvements in grip strength, swelling reduction, and changes in Spurling test results were observed. However, the patient reported increased tenderness in the right lateral epicondyle. Consequently, the treatment plan was revised to include radial nerve entrapment management, with phase 2 initiated independently of phase 1. Ultimately, the patient was treated with DCS, which addressed both the proximal cervical radiculopathy and distal radial nerve compression. The treatment consisted of acupuncture, pharmacopuncture, Chuna manual therapy, and taping as part of an integrative TKM approach.

Through acupuncture treatment, phase 1 focused on regulating qi and blood in the C-spine and shoulder girdle. Phase 2 expanded the treatment to include additional points in the meridian of the large intestine and tender points near the right lateral epicondyle to manage pain. Pharmacopuncture in phase 1 involved using a Shinbaro pharmacopuncture solution, commonly used for cervical disc-related conditions, injected near the C6/7 facet joint.^[19]^ In phase 2, an additional injection was administered at a tender point near the elbow to address peripheral nerve entrapment.^[9]^ The Chuna manual therapy incorporates prone and supine cervical correction techniques, supine cervical JS stretching correction, and supine sternoclavicular correction to address cervical misalignment, restore function, and improve ROM. Furthermore, based on a previous case study,^[20]^ taping was introduced in phase 2 to reduce tenderness and alleviate discomfort in daily activities caused by radial nerve entrapment.

DCS has not been widely investigated in Korea, and its diagnosis can be challenging owing to symptom overlap with cervical radiculopathy-associated radiating pain. In this case, the presence of hand edema (an uncommon feature of cervical radiculopathy) raised the suspicion of peripheral nerve entrapment. Further findings, including tenderness over the right lateral epicondyle and positive ULTT findings, confirmed the diagnosis of radial nerve entrapment, allowing treatment from the perspective of DCS. A phased treatment approach observed significant improvements in the patient’s NRS score, grip strength, pitting edema grade, tenderness over the right lateral epicondyle, Spurling test findings, and ROM normalization. These results clinically validate the effectiveness of the 2-phase treatment strategy.

## 4. Conclusions

In the present case, the patient visited our hospital with pain radiating from the right posterior cervical region to the trapezius and medial scapular border, along with paraesthesia extending from the right forearm to the fingers. These symptoms followed external trauma near the thoracic spine and scapular area on October 13, 2023. The patient underwent TKM treatment from the perspective of DCS for 8 weeks (October 27–December 20, 2023), resulting in the following outcomes: First, this case approached cervical radiculopathy within the DCS framework with a TKM treatment plan, an area with limited research in South Korea currently. Second, the phased treatment approach facilitated the sequential and comprehensive management of cervical radiculopathy and peripheral nerve entrapment from the perspective of DCS. Third, this phased approach highlights the clinical efficacy of TKM in managing DCS.

However, in the diagnosis of DCS, although cervical radiculopathy was accurately confirmed via C-spine MRI, radial nerve entrapment was determined solely based on physical examination and clinical symptoms. Given the current context in which ultrasonography is considered one of the most effective tools for evaluating and imaging peripheral nerve-related pathology,^[21]^ the incorporation of ultrasound diagnostics could improve the accuracy of identifying nerve entrapment sites and the effectiveness of Korean medicine treatments for DCS.

Future studies should include multiple case reports using ultrasound, comparisons of the efficacy of single and integrative treatments, and broader clinical studies to refine the approach proposed in this case report. Such studies could contribute to a more comprehensive understanding and validation of TKM’s role of TKM in DCS treatment.

## Author contributions

**Conceptualization:** So Rim Kim, Yeon Sun Lee, Jin Hyun Kim, Min Jo Seo.

**Data curation:** So Rim Kim, Yeon Sun Lee.

**Investigation:** So Rim Kim.

**Supervision:** Yoon Jae Lee, In-Hyuk Ha.

**Writing – original draft:** So Rim Kim.

**Writing – review & editing:** Sook-Hyun Lee, Yoon Jae Lee, In-Hyuk Ha.
